# Relapse Prevention Intervention after Suicidal Event (RISE): Feasibility study of a psychotherapeutic short-term program for inpatients after a recent suicide attempt

**DOI:** 10.3389/fpsyt.2022.937527

**Published:** 2022-07-22

**Authors:** Lydia Bahlmann, Marlehn B. J. S. Lübbert, Thomas Sobanski, Ulrich W. Kastner, Martin Walter, Stefan Smesny, Gerd Wagner

**Affiliations:** ^1^Department of Psychiatry and Psychotherapy, Jena University Hospital, Jena, Germany; ^2^Department of Psychiatry and Psychotherapy, University of Rostock, Rostock, Germany; ^3^Department of Psychiatry, Psychotherapy, and Psychosomatic Medicine, Thüringen-Kliniken, Saalfeld, Germany; ^4^Department of Psychiatry and Psychotherapy, Helios Fachkliniken Hildburghausen, Hildburghausen, Germany

**Keywords:** suicidal behavior, suicide re-attempts, self-harm, self-injury, cognitive-behavioral therapy, suicide prevention, psychotherapy, RISE

## Abstract

Recent research suggests that treating only mental disorders may not be sufficient to reduce the risk for future suicidal behavior in patients with a suicide attempt(s). It is therefore necessary to pay special therapeutic attention to past suicidal acts. Thus, the newly developed RISE (Relapse Prevention Intervention after Suicidal Event) program was built on the most effective components of existing psychotherapeutic and psychosocial interventions according to our current meta-analysis. The RISE program consists of five individual sessions designed for the acute psychiatric inpatient setting. The main goals of the treatment are to decrease future suicidal events and to improve patients' ability to cope with future suicidal crises. In the present study, feasibility and acceptance of the RISE program were investigated as well as its clinical effects on suicidal ideations, mental pain, self-efficacy and depressive symptoms. We recruited a sample of 27 inpatients of the Department of Psychiatry and Psychotherapy, University Hospital Jena, Germany. The final sample consisted of 20 patients hospitalized for a recent suicide attempt, including 60 percent of multiple attempters. The data collection included a structured interview and a comprehensive battery of questionnaires to evaluate the feasibility and acceptance of the RISE program as well as associated changes in clinical symptoms. A follow-up examination was carried out after 6 months. Considering the low dropout rate and the overall positive evaluation, the RISE program was highly accepted in a sample of severely impaired patients. The present study also demonstrated that the levels of suicidal ideations, mental pain, depressive symptoms, and hopelessness decreased significantly after RISE. Since all of these clinical parameters are associated with the risk of future suicidal behavior, a potential suicide-preventive effect of the intervention can be inferred from the present findings. The positive results of the follow-up assessment after 6 months point in the same direction. In addition, RISE treatment increased self-efficacy in patients, which is an important contributor for better coping with future suicidal crises. Thus, present study demonstrate that RISE is a suitable therapy program for the treatment of patients at high risk for suicidal behavior in an acute inpatient setting.

## Introduction

World-wide ~800,000 individuals die each year from suicide and ten to twenty times more attempt suicide (1), thus having a devastating impact on relatives, friends, and ultimately society as a whole. In addition, the widespread availability of the Internet contributes to the increasing awareness of suicide methods increasing thereby the risk for suicidal behavior (2, 3). Current suicide research suggests that the development of suicidal ideation (SI) and the transition from ideation to a suicidal act are distinct phenomena with distinctive explanations and predictors (4, 5). For example, Major Depressive Disorder (MDD) has been considered as one of the most significant risk factors for a long time. However, the clinical significance of this association has recently been questioned, since MDD facilitates the development of SI, but does not allow for the prediction of suicidal behavior or the distinction between comparatively harmless SI and the much more critical suicidal behavior (SB) (4–6). On the other hand, more brain abnormalities in terms of altered white matter integrity have been reported in children and adolescents with bipolar than in those with unipolar disorder (7), being associated with more adverse outcomes, like SB (8).

In the fifth edition of Diagnostic and Statistical Manual of Mental Disorders (DSM-5) criteria were therefore proposed for a “suicidal behavior disorder” (SBD) as a “condition for further study,” thus considering it as a possible category of its own and not solely as a symptom of a mental disorder. Consequently, the sole focus on treatment of MDD or other mental disorder may not be sufficient to reduce the risk for future SB, even if demonstrating effectiveness in reducing SI (9).

Previous suicide attempts (SAs) are judged as the most important predictor of future suicide and suicide attempts (10). There is a substantial risk of dying by a subsequent attempt for individuals surviving an index attempt (11, 12). Moreover, a recent meta-analysis has clearly demonstrated that in the first 3 month after discharge from psychiatric facilities the suicide rate is approximately 100 times higher than the global suicide rate, in particular in patients admitted after suicide attempt (13). Therefore, these findings underscore the urgent need for treatment strategies specifically tailored for individuals with previous suicide attempts to reduce the risk of suicide re-attempts. In addition, due to relatively low outpatient treatment adherence in these patient group (14), a timely intervention immediately after admission is highly desirable to improve help seeking behavior (15).

Previous systematic reviews investigated the efficacy of prevention strategies by combining psychosocial, such as telephone contact, postcards, and case management and specific psychotherapeutic treatments. However, mixed findings were reported regarding suicide re-attempts (16, 17). In our recent meta-analysis and systematic review (18) we solely focused on psychotherapeutic interventions to reduce suicide re-attempts, which included patients with a suicide attempt as defined by DSM-5. Our main finding was that cognitive behavioral therapy (CBT) based interventions were significantly more efficacious than the applied control conditions in reducing the number of suicide re-attempts in the investigated follow-up period. The CBT trials specifically aimed preventing patients to slide into the so-called “suicidal mode” (19), in which the range of functional problem solutions is dramatically reduced. Thus, laying the psychotherapeutic focus on suicidal episodes might be the key intervention for preventing suicide reattempts and suicide.

Furthermore, a recent randomized controlled trial demonstrated promising effects of acceptance and commitment therapy (ACT), i.e., a development of the so-called “third wave” of behavioral therapy, on the reduction of suicidal ideation in patients suffering from SBD according to DSM-5 (20). The authors reported that ACT was more effective than a relaxation control condition in reduction of the severity and intensity of suicidal ideation as assessed with Columbia Suicide Severity Rating Scale (C-SSRS). This effect remained significant after 3-month follow-up. Furthermore, significantly decreased levels of hopelessness and psychological pain were observed after ACT compared to the control condition. Finally, a further promising brief psychosocial intervention to reduce future suicide risk is the Safety Planning Intervention (SPI), originally published by Stanley and Brown (21). The safety plan includes, for example, recognizing warning signs and antecedents of a suicidal crisis and utilizing internal coping strategies. In a recent study, Stanley et al. (15) demonstrated that patients in the emergency departments receiving SPI condition were less likely to engage in suicidal behavior than those receiving usual care during the 6-month follow-up period.

Thus, in the newly developed Relapse Prevention Intervention after Suicidal Event (RISE) psychotherapeutic program, we selected key elements of the previous CBT interventions based on our meta-analysis (18). The core overlapping elements of these interventions were (1) helping the individual to detect and understand the triggering conditions for one's prior suicide attempt(s), e.g., in terms of a cognitive-behavioral case conceptualization; (2) training the individual specific strategies for preventing and managing a future suicidal crisis; and (3) testing the individual to manage future suicidal crises by employing a relapse prevention task (22–24). Thus, these interventions specifically aim to prevent an individual to slide into the “suicidal mode” by providing tailored strategies to manage future suicidal crises, for example *via* a safety plan. Therefore, we also integrated the psychosocial SPI intervention (15). Furthermore, suicidal thoughts usually precede suicidal behavior. Suicidal individuals report automatic and intrusive ideas and mental images of suicide, often experienced as uncontrolled, which they try to suppress, increasing thereby their salience and frequency (25). Also, the stigma associated with suicidal ideas hinders individuals to manage such thoughts in a functional way (26). Thus, providing psychoeducational information and practicing specific strategies to cope with suicidal ideas and urges is considered an effective way of preventing suicidal behavior (27). Specific techniques of the ACT were shown to be effective in managing and reducing suicidal ideation (20). We therefore included these techniques into the RISE program.

Moreover, understanding the course of a suicidal crisis (session 1) as well as identification of individual triggers for the suicidal crisis in the session 2 (case conception) are necessary prerequisites for the development of a comprehensive and feasible safety plan. In addition, both the psychoeducational intervention (Session 3) as well as the structured homework outside the sessions provide important information to develop together with the therapist appropriate strategies for coping with future suicidal crises. Making a safety plan without preparatory work could lead to a more superficial information in the safety plan and, consequently, to less effective strategies.

Therefore, RISE consists of five ~50-min sessions (totaling in ~5 h) of individual psychotherapy delivered over the course of 2 to 3 weeks during inpatient hospitalization after a suicide attempt. Furthermore, the five-session structure was chosen to allow the RISE program to be finished in the acute psychiatric inpatient setting.

Providing a brief, structured, feasible and potent psychotherapeutic intervention already during acute inpatient care to individuals at high risk for future SB aims to reduce the risk for suicide re-attempts, to increase capability to cope with suicidal ideation and future crises as well as to increase treatment motivation for subsequent treatment.

The main objective of this open-label pilot study was to assess the feasibility and the acceptance of the RISE program in a limited number of patients administered to an inpatient care after a suicide attempt. Based on previous publications, we hypothesized high acceptability of the RISE treatment. We also hypothesized that after RISE, patients would exhibit reduced suicidal ideation, reduced levels of psychological pain and hopelessness. We also expected to find an increase in self-efficacy after RISE treatment in managing future crises.

## Methods

### Study participants

**Figure 1:**
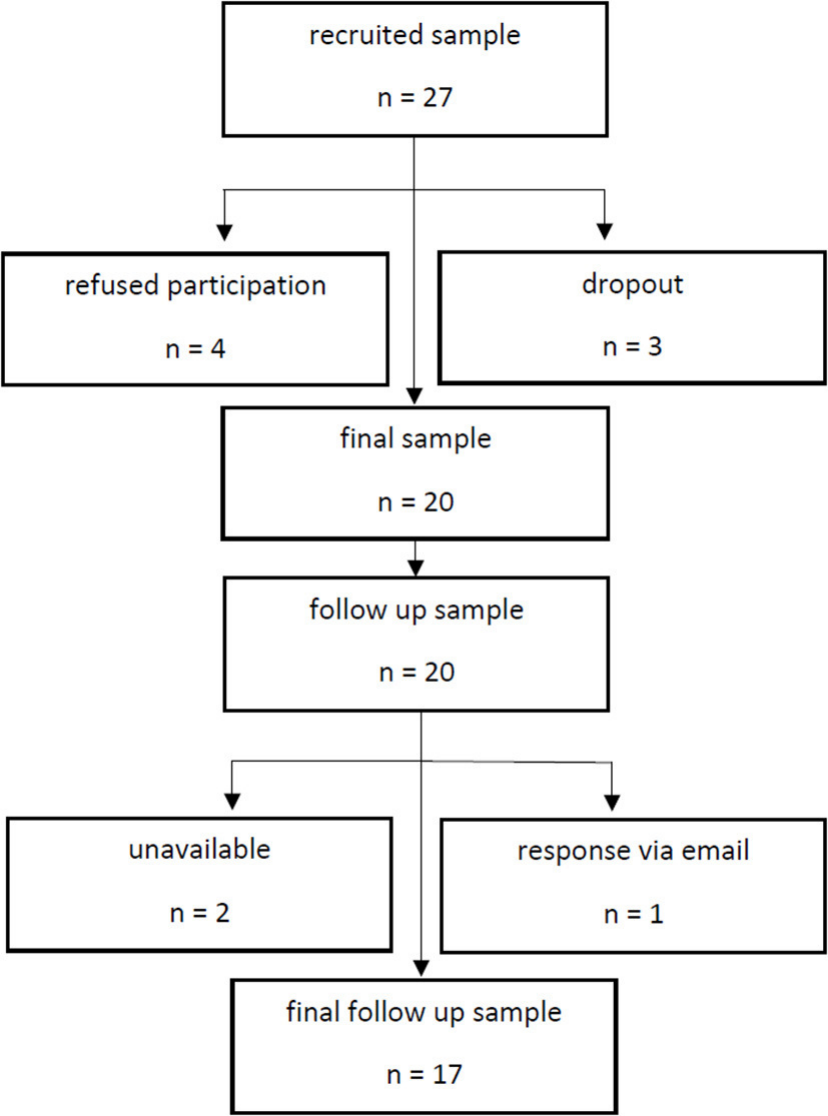
Study flow chart.

As illustrated in Figure 1, we recruited a sample of 27 adults from the inpatient ward of the Department of Psychiatry and Psychotherapy, University Hospital Jena, Germany from September 2020 to May 2021. The study was part of an ongoing suicide prevention project [“Network for Suicide Prevention in Thuringia (NeST)”], founded by the Federal Ministry of Health (Bundesministerium für Gesundheit, BMG). NeST is based on the cooperation of three psychiatric clinics in the federal state Thuringia. The goal of the NeST is to implement suicide prevention actions in Thuringia using evidence-based multi-level interventions.

All patients were hospitalized in a psychiatric ward after a recent suicide attempt. The inclusion criterion was the fulfillment of the DSM-5 criteria for current suicidal behavior disorder (SBD), which is explicitly defined as “a self-initiated sequence of behaviors by an individual who, at the time of initiation, expected that the set of actions would lead to his or her own death” (28). Since this definition strongly emphasizes the intent to die, we have systematically assessed it by means of the Suicide Intent Scale, SIS (29) in our study. SIS is dealing with both the circumstances of a suicidal act and patient's self-report on it. Exclusion criteria were acute psychosis, acute intoxication, or withdrawal symptoms, diagnosed intelligence impairment, language barriers, lack of insight and dementia.

Four patients refused to participate in the RISE program. Two patients dropped out after the initial interview because of hospital discharge. In one patient the program was discontinued due an acute suicidal crisis. Thus, the final sample consisted of twenty patients, whereby fifteen of the patients (75%) had a diagnosis of MDD. In addition, one patient fulfilled the ICD-10 criteria of alcohol dependence, two patients met obsessive-compulsive disorder (OCD) criteria, one patient had an acute stress disorder (ASD) and one patient bulimia nervosa (BN). Most of the included patients were multiple attempters, i.e., twelve of twenty patients (60%) had at least one past suicide attempt and eight of them two or more attempts. Further demographic and clinical information are depicted in Table 1.

**Table 1:** Sociodemographic and suicide attempt related characteristics of the final sample.

**Characteristic**	**Patients (*****n*** **=** **20)**
Gender (% male)	11 (55%)
Age (years) (Mean ± SD)	35.6 ± 14.2
Suicide intent scale (Mean ± SD)	12.5 ± 4.3
Education	
10 years school	8 (40%)
12 years school	11 (55%)
University/college	1 (5%)
Family status	
Unmarried/ no partner	14 (70%)
Married/ in relationship	6 (30%)
Employed (%)	14 (70%)
Living alone (%)	8 (40%)
Index suicide attempt
*X60–X64: drug intoxication*	13 (65%)
* X78: Self-harm from a sharp object*	4 (20%)
*X65: Self-poisoning from alcohol*	1 (5%)
*X70: Self-harm from strangulation*	1 (5%)
*X82: Self-harm from motor vehicle accident*	1 (5%)

Eligible patients were initially interviewed within the first week after the suicide attempt by a trained psychologist (M.L., L.B.). All therapists had a master's degree in psychology and were currently in CBT psychotherapist training. In addition, there was a weekly supervision by an experienced licensed psychotherapist (G.W.).

In a 60-min introductory interview the RISE therapy program was explained in detail to the study participants. They were asked to review and sign an informed consent form regarding this study, which was approved by the ethics committee of the Friedrich-Schiller University Jena, Germany. Furthermore, baseline demographic and clinical data were assessed during this interview. Subsequently, self-report questionnaires were explained and given to the patients.

### Clinical assessment

The data collection included a structured interview and a comprehensive battery of questionnaires to evaluate the feasibility and acceptance of the RISE program as well as associated changes in clinical symptoms. To assess the feasibility of the treatment, patients were asked about their experience with the treatment in general, the applied treatment techniques and the perceived acceptability of RISE using an exit survey. The questionnaire consisted of 10 open-ended questions designed by the study team. We were particularly interested in evaluating patient's experience with the intervention and in identifying potential difficulties with the treatment, as provided in Table 2.

**Table 2:** Questions regarding the RISE feasibility.

1. Can you tell us what you think about the RISE psychotherapeutic program in a few words?
2. What changes have you noticed since the start of the treatment?
3. How did you benefit from the therapy? What do you attribute that to?
4. Is there anything about the therapy program that you have found difficult or even harmful? Can you tell us more about it?
5. Please indicate the parts of the program that have troubled you. Please indicate how and when you experienced this.
6. Please state the situations / exercises in the program that had a relieving or calming effect on you. Please indicate how and when you experienced the relief.
7. If a friend was in your situation, would you recommend them to take part in the RISE program? Why or why not?
8. What else would you like to tell us?
9. On a scale from 1 to 5 (1 = not at all; 5 = several times a day), how often have you used the techniques you have learned to help you distance yourself from (suicidal) thoughts and to cope with difficult situations, thoughts, or emotions within the last few weeks?
10. On a scale from 1 to 5 (1 = not at all; 5 = several times a day), how often have you used the Safety Plan to cope with difficult situations, thoughts or emotions within the last few weeks?

Suicidal ideations before and after RISE treatment were assessed using the German version of the Beck Scale for Suicide Ideation, BSS (30). The BSS is a 19-item self-report scale for measuring patient's thoughts, plans and intent to commit suicide. The items are rated on a three-point scale from 0 to 2. Total scores range from 0 to 38, higher scores indicate more severe suicidal tendencies. Furthermore, at the beginning of the first, third and last RISE session we used a numeric scale from 1 to 10 to assess the intensity of SI and the intent to act on SI. We also used the numeric scale from 1 to 10 to measure the intensity of psychological pain before those RISE session. Pre- and post-treatment depressive symptoms were evaluated by the clinician-rated Montgomery-Åsberg Depression Rating Scale, MADRS (31) and by the self-rated Quick Inventory of Depressive Symptomatology-Self Rating, QIDS-SR (32). The QIDS-SR is a brief 16-item questionnaire assessing the severity of depressive symptoms within the past week. The total score ranges between 0 and 27, with higher scores indicating more severe depressive symptoms. Hopelessness and pessimism concerning the future was measured with the revised version of the validated German Hopelessness Scale, BHS (33), based on Beck's cognitive theory of depression (19). The General Self-Efficacy Scale (SWE) was used to assess changes in patient's self-efficacy before and after treatment, i.e., in the belief that one's actions are responsible for successful outcomes (34). The scale consists of 10 items to rate general, optimistic self-beliefs, i.e., the confidence to cope with a difficult situation, whereby the success is attributed to one's own competence. To evaluate the quality of the patient–therapist relationship, we used the 11-item self-reported Helping Alliance Questionnaire, HAQ (35). In addition to the general sum scale, two subscales measure patient's relationship satisfaction and patient's experienced treatment success.

### Description of the RISE program

The RISE program is a brief structured psychotherapeutic intervention primarily developed for patients admitted to an inpatient unit after a suicide attempt. The main goal of the treatment is to reduce the likelihood of future suicidal events and to increase patient's ability to effectively cope with future suicidal crises. Therefore, it focuses on the following objectives: increasing crisis expertise, developing a line of actions for crisis management, and empowering the patient to manage suicidal ideation. Furthermore, due to the condensed structure of the RISE program it is specifically designed for the implementation in a psychiatric hospital. After baseline assessment, patients receive 5 treatment sessions with different key topics. The sessions last ~50 to 60 min and are conducted two to three times per week to complete the program within 2–3 weeks. In some cases, sessions like “behavioral analysis” had to be extended up to 90 min.

All sessions build on each other so that a deeper understanding of triggers and causes of the suicidal crisis can be generated in both therapist and patient. In this way, more profound and individualized strategies can be developed in the safety plan, which in turn leads to better prevention of future suicidal crises. In addition, the sessions are accompanied by various worksheets and exercises between sessions to deepen the understanding of the topic, to promote introspection skills and enabling to identify and discuss emerging problems. Thereby, it is recommended to follow the given format. Furthermore, the five-session structure was chosen to allow the RISE program to be finished in the acute psychiatric inpatient setting.

RISE follows a specific session structure. Each session is framed by a brief mood and arousal assessment as well as agenda setting, homework review and sessions summary at the end. Furthermore, at the beginning of each session the frequency, duration and intensity of suicidal thoughts since the last session are assessed.

#### Session 1

##### Behavioral analysis

The first session of the program focuses on the last suicide attempt. The aim is to generate a detailed timeline from the beginning of a suicidal crisis till the recent suicide attempt, to identify individual warning signs and triggering events. The behavioral chain analysis is used as a technique to reveal patient's thoughts and feelings leading to SA, to help patient to identify “the point of no return” during the analysis. This approach was selected to assess individual's reaction during the suicidal crisis at different levels, i.e., thoughts, feelings, body sensations, and behavior and visualizing them as a behavior chain. At the end of the session, patients are provided with information about the development of suicidal crises and are encouraged to elaborate warning signs as homework.

#### Session 2

##### Case conceptualization

In the second session, the therapist develops an individual case conceptualization based on the behavioral chain analysis. The goal of this session is to produce a deeper understanding of suicidal behavior. Besides personal triggers, the therapist helps to identify acute and biographical stressors as well. Protective factors are also explored for resource activation and to achieve a relieving effect. As homework, patients are asked to make a list of current incriminating thoughts that will be addressed in the next session.

#### Session 3

##### Psychoeducation and managing suicidal ideation

The third session of the program focuses on patient's suicidal ideations and on strategies to cope with them. The aim is to increase the patient's understanding regarding triggers for the emergence of suicidal thoughts, different levels of the intensity as well as their function. The defusion technique from the ACT is used as a tool to gain distance from suicidal thoughts and thus to cope with them (36). The judgment-free acceptance of the emergence of suicidal thoughts is practiced. The defusion of incriminating thoughts is achieved through various methods, ranging from cognitive approaches, such as counting out and rearranging thoughts written in sentences, to imagination exercises for distancing, to humorous approaches, such as singing out the content. As homework, patients should practice the defusion techniques they have learned and systematically record the effectiveness of those strategies. In addition, it is an important goal of this session that the participants learn to detect the point at which suicidal thoughts might become life-threatening.

#### Session 4

##### Safety planning

The aim of the fourth session is to create an individual safety plan. Within the first step, the therapist helps the patient to identify and list individual early warning signs and antecedents of a suicidal crisis. Several internal coping strategies and social contacts are collected to distract from suicidal thoughts, including reaching out to suitable family members and friends. Besides, the therapist helps the patient to identify and list mental health professionals, agencies, or emergency services. Moreover, concrete steps are discussed to restrict access to means during the beginning of a suicidal crisis. As homework, patients are instructed to prepare a “Hope Box” for the next session, which contains their personal reasons for living and reminds them, e.g., by means of photos, postcards or quotes. Also helpful tools from therapy can also be stored in the Hope Box, such as written imagination exercises.

#### Session 5

##### Relapse prevention

In the last session of the program, the safety plan is reviewed and expanded based on the last session and patient's homework. Using the behavioral chain analysis from the first session, the developed safety plan is applied to the latest suicidal crisis. The patient and the therapist review the behavioral analysis step by step to test the likelihood of use and effectiveness of the acquired skills and coping strategies in preventing future suicidal behavior. The therapist helps the patient to identify suitable strategies and re-examines how to increase the likelihood to apply these strategies. As a final homework assignment, patients are asked to implement the safety plan in their home environment.

### Letter intervention

After finishing the five RISE sessions, patients are contacted by sending a letter after discharge during the follow-up period of twelve month, as proven to be an effective preventive intervention by Motto and Bostrom (37). In addition to enhance the connectedness with the patient, the further aim is to remind patients of key elements of the RISE program to re-activate the learned skills. To reach this aim, four personalized letters are dispatched in the first year after the end of RISE treatment, containing reminders of the strategies that were practiced within the program as well as of the possibilities for professional crisis support in case of an acute suicidal crisis. During the 6-month follow-up period, we received responses from eight patients (40%), only one of our participants refused to get contacted by letter.

### Treatment as usual

Besides the participation in the RISE program patients received a treatment as usual (TAU). TAU includes an individual session with a psychologist once a week lasting 50 min. The focus on suicidal behavior of the patients was exclusively laid within the RISE program. Most of the study participants additionally received psychopharmacological medication, fourteen patients (70%) were medicated with antidepressants and seven of them (35%) also received antipsychotics. Six patients (30%) were free of any psychopharmacological medication.

### Follow-up

As part of a follow-up investigation approximately 6 months after completion of the RISE, we conducted telephone interviews with the study participants. The aim was to obtain information about the potential occurrence of suicidal behavior. Furthermore, we explored to what extent patients applied specific RISE program elements, e.g., the safety plan within their everyday life.

### Statistical analysis

We used SPSS Version 26.0 (https://www.ibm.com/de-de/analytics/spss-statistics-software) for the statistical analyses. To investigate the pre-post differences in clinical scales (e.g., suicidal ideation, hopelessness, depression) within-subjects ANOVA and student's paired *t*-tests were calculated based on the data of clinical questionnaires.

## Results

### Feasibility and acceptance

Regarding treatment adherence all but one patient (95%) completed the program. Only one patient discontinued RISE due to an acute suicidal crisis. Patient's responses to the open-ended survey questions were summarized by using techniques of the qualitative content analysis (38) to identify major topics (see Table 3). The responses were categorized through systematical reduction and filtering out overlapping content between individual patient statements. Double coding within a patient statement was possible because some statements contained multiple key points.

**Table 3:** Categorization of patients's feedback regarding RISE feasibility.

**Topic**	**Subtopic**	**Coding results**	**Quote**
Global evaluation		Helpful	“[RISE is] very helpful, intensive crisis preparation to feel safer after discharge. Competent, friendly support, [I am] glad that I participated in the program.”
		Informative	“[The program is] informative, [it] brings self-knowledge and new perspectives.”
		Intense	“[A] very intensive and effective program.”
Positive effects	Improvements	Less hopelessness and improved life prospects	“After winter comes spring. [I have] more hope for improvement.”
		More self-effectiveness	“[I am] more confident in dealing with my depression and [I] know what to do in suicidal situations.”
		Less suicidal ideations	“I have taken more distance from suicidal thoughts.”
	Relieving aspects	Therapeutic alliance	“[The] intensive therapeutic talks helped a lot. They showed me that the future has many beautiful moments in store.”
		Defusion techniques	“During the session applying defusion techniques, I felt relief.”
		Safety plan	“I got more stability with concrete plans to ensure my safety at home.”
Negative effects	Challenging aspects	Behavioral analysis	“The re-experience of the suicide attempt upset me because I had to ‘live through' that moment again.”
		Incriminating thoughts	“[It was] difficult to apply [defusion] techniques at first, as incrementing thoughts were very present and strong.”
		Intensity	“[(RISE was] not harmful, but in retrospect, the sessions sometimes overwhelmed me.”

The data was coded and recoded by two research team members (L.B.; G.W.). When coding was not consistent between coders, final codes were determined by consensus. To simplify the presentation of results, statements to questions 1, 7, and 8 (Table 2) were subsumed into the global assessment of the program. Similarly, responses to questions 2, 3, 4, 5, and 6 (Table 2) were used to filter positive and negative aspects of the program.

Regarding the global evaluation of the program, there was no negative feedback from patients. In more detail, 18 out of 20 (90%) emphasized positive aspects of RISE in the overall evaluation. More specifically, seventeen patients evaluated the RISE program as helpful (85%). Eight patients (40%) stated that the content of the sessions was informative, because they could increase their knowledge about suicidal ideations and behavior and how to deal with that. Regarding the main improvements, nine patients (45%) reported less hopelessness and improved life prospects as the main positive outcome of the treatment. Furthermore, eight participants (40%) reported more self-effectiveness in managing future suicidal crises and four patients (20%) highlighted decreased intensity of suicidal ideations as the main improvement of the sessions.

In more detail, for most of the patients (80%) the therapeutic alliance and the possibility to talk about SI was considered as the main relieving aspect of the program. The safety pan was emphasized by 10 patients (50%) and ACT defusion techniques by 11 patients (55%) as main relieving components of the RISE program. The hope box was mentioned by two patients (10%) as a helpful component of the program.

Regarding the negative aspects or strains during or after the program, 11 participants (55%) did not report any difficulties with RISE. None of the patients considered any part of the program as harmful but some challenging aspects were acknowledged. The behavioral analysis was considered as demanding by sixteen patients (80%). Four participants (20%) indicated that the RISE program was intense regarding the workload of the program and its focus on the SA. For three patients (15%) exposure to suicidal thoughts was challenging and further three patients (15%) described the program as intense. Additionally, two patients (10%) experienced incriminating memories during the case conceptualization and one patient (5%) considered the creation of the safety plan as rather difficult task. Two participants (20%) indicated no changes at all during the treatment. Table 3 provides a summary of the categorized patient's feedback including participant's quotes.

Patients indicated on a scale from 1 to 5 that they have applied learned methods to gain distance to suicidal thoughts (M = 3.00; SD = 1.21) and techniques from the safety plan (M = 2.35, SD = 1.04) few to several times in an inpatient unit.

### Effects of the RISE program on clinical symptoms

Regarding suicidal ideations, a significant reduction was found in the BSS after RISE intervention [M_0_ = 12.1 ± 9.75, M_1_ = 7.7 ± 9.14: t (df = 19) = 3.25, *p* = 0.004; Cohen's d = 0.75]. Using within-subjects ANOVA with the NAS scores over 3 times (session #1, #3 and #5), we did not observe any significant changes in SI intensity or in the intent to act on SI, which might be due to a low intial scores in the NAS. But, a highly significant decrease in the intensity of mental pain was detected [M_0_= 4.3 ± 3.06, M_1_= 3.1 ± 2.36, M_2_= 2.7 ± 2.64: *F*_(2, 38)_ = 5.4, *p* = 0.009; partial η^2^ = 0.22; Cohen's f = 0.53]. Regarding depressive symptoms, we found a highly significant reduction in clinician-rated [M_0_ = 19.9 ± 10.21, M_1_ = 13.1 ± 11.27: MADRS: t (df = 19) = 3.53, *p* = 0.002; Cohen's d = 0.79] and self-reported depressive symptoms [M_0_ = 12.2 ± 5.16, M_1_ = 7.2 ± 5.20: QIDS: t (df = 18) = 5.10, *p* < 0.001; Cohen's d = 1.17] after RISE program. We also observed a significant reduction in the levels of hopelessness as assessed by the German Hopelessness Scale [M_0_ = 77.3 ± 19.14, M_1_ = 56.63 ± 21.49: t (df = 18) = 4.131, *p* = 0.001; Cohen's d = 0.95]. Finally, in the self-efficacy expectancy scale, which measures patient's belief of being able to successfully cope with critical challenging situations on one's own, we found a highly significant increase in the sum score after RISE [M_0_ = 22.4 ± 7.49, M_1_ = 27.1 ± 5.66: t (df = 19) = −4.14, *p* < 0.001; Cohen's d = 0.93]. This change in the self-efficacy indicates improved coping expectations of future crises. Regarding the quality of the therapeutic relationship, patients indicated in the HAQ high degree of patient–therapist relationship satisfaction (M = 32.5 ± 3.8) and the treatment success satisfaction (M = 24.3 ± 4.5).

### Follow-up

Regarding the follow-up investigation after 6 months, we could reach 18 of 20 patients (90%) by phone. One of these patients declined the telephone interview but provided feedback *via* e-mail. Sixteen patients (89%) reported not having any suicide re-attempts. Two patients, who already had a history of three and four suicide attempts, respectively, reported a suicide re-attempt within the follow-up period. All contacted patients reported that they had used the safety plan at least partly. Six patients (35%) revised the safety plan during the follow up period. Most of the patients stated that they have used coping strategies (94%), i.e., utilizing internal coping strategies and outreach to social contacts to distract from suicidal thoughts. Furthermore, a majority reported that they have sought professional help (82%) and engaged their social environment for the safety planning (65%). Furthermore, some patients listed the hope box (65%) and ACT defusion techniques (53%) as additional strategies used.

## Discussion

RISE is a brief and structured psychotherapeutic intervention specifically designed for patients after a suicide attempt in an acute psychiatric inpatient setting with the goal to prevent future suicidal behavior. The RISE program was created after systematically reviewing studies on previous psychotherapeutic and psychosocial interventions for suicidal behavior (18, 20, 21). In the present study, feasibility and acceptance of RISE program were investigated as well as its effect on suicidal ideations, mental pain, self-efficacy and depressive symptoms.

The results of the feasibility questionnaire along with the treatment adherence demonstrate that RISE is a feasible intervention which is also highly accepted by the patients. Although most participants were multiple attempters, the patient commitment was remarkably high. The treatment adherence within these patient population is known to be low-more than 50% of patients drop out after the first session (39). Compared to that, only few patients refused the participation in the RISE program and only one patient discontinued the treatment because of an acute suicidal crisis. The high adherence may be due to the high structuredness of the RISE program and its timely start immediately after the psychiatric admission due to a suicide attempt. Stanley et al. (15, 40) recommended a timely intervention and more frequent number of sessions at the beginning of the treatment to improve help seeking behavior. The authors also emphasized the benefits of a limited number of sessions focusing on specific skills to deal with a future suicidal crisis.

Overall, the vast majority of patients have evaluated the RISE program as helpful. There was no negative feedback regarding the global evaluation. The sound therapeutic alliance, but also ACT defusion techniques and the safety planning were considered as the most relieving factors. Based on our impression, patients experienced especially the session #3 on dealing with suicidal thoughts as relieving and depathologizing. In addition, many patients indicated that they felt more confident in dealing with crises because of having the safety plan available. Only few patients described RISE as intense related to the exclusive focus on SB during the program. Most of our patients did not report any negative experiences with RISE, even if the behavioral analysis was evaluated as challenging. It was important that we allowed sufficient time in the behavioral analysis session to be able to deal with potentially triggered negative affect. Additionally, the emergence of incriminating thoughts and the intensity of the program were considered as challenging by some patients. Nevertheless, all but one patient completed the program despite these challenging aspects and the dense structure.

Investigating changes in specific clinical variables, our results show that the RISE program may reduce the risk for future suicidal behavior through several factors. A significant reduction of suicidal ideations, in the intensity of mental pain, depressive symptoms and hopelessness was observed after RISE treatment. Importantly, patients reported a significantly higher degree of self-efficacy after the intervention. In particular, the reduction in the intensity of mental pain, as a potentially specific marker of suicidal risk, may represent a promising aspect of treatment success.

The finding that only 20% of the patients emphasized in the open-ended survey questions the reduction of suicidal ideation as a main improvement may be considered as unexpected, at first glance. However, since most of our patients had at the beginning of the RISE intervention relatively low level of suicidal ideations, it is conceivable that the reduction of suicidal ideas was not considered the most important treatment goal by patients. It was rather regaining a perspective for the future and increasing self-efficacy to deal with future suicidal crises.

In addition, systematic reviews consistently reported that across a range of therapies and diagnoses a good therapeutic alliance predicts positive treatment outcome (41, 42). Specifically, a strong therapeutic relationship appears to play a major role for a successful treatment of patients after a suicide attempt (40). We therefore placed special emphasis on the “therapist variable,” who have to demonstrate empathetic, collaborative, and non-judgmental stance and, thus, enabling the patient to openly talk about suicidal ideations and behavior. Patients' reports confirmed the importance of laying a specific focus of RISE on a sound therapeutic relationship, since it was considered as the main relieving aspect by most of our participants and further supported by the high patient–therapist relationship satisfaction scores in the HAQ questionnaire. Furthermore, the structured and manualized approach of RISE enables even less experienced therapists to carry out the treatment by clear instructions for each session.

Regarding the results of the follow-up assessment after 6 months most of the contacted patients have not attempted suicide again. But two patients (11%) had a suicide re-attempt in the follow-up period. It is well known that a history of previous suicide attempt is the strongest predictor for future suicide attempts. Both patients had a history of multiple attempts. This number is similar or even lower compared to the ratios of suicide re-attempts after psychotherapeutic interventions as reported in previous studies (18). Furthermore, considering that our sample included 60 percent of multiple suicide attempters, the present result that nearly 90 percent of patients have not committed or attempted suicide during the 6 months after RISE treatment is promising.

In addition, all patients used the learned strategies like the safety planning at least partly at their home environment. It is also noteworthy that most patients were willing to seek professional help and treatment after RISE treatment and involved their social environment in managing their future suicidal crises. In addition, most patients applied learned coping strategies to manage a newly beginning suicidal crisis. This finding corresponds to a significant increase in the degree of self-efficacy after RISE therapy, which is an important factor for feeling less hopeless and effectively coping with stressful life events.

Alternative to the SPI, Crisis Response Planning (CRP) has been also shown to be effective on reduction of inpatient hospitalizations and suicidal ideations in U.S. army soldiers compared to the contract for safety (43). Like SPI, personal warning signs, coping strategies, social and professional crisis support as well as individual reasons for living are outlined. Thus, it would be also conceivable to add the CRP in future implementation of the RISE program.

This study has some limitations. Since the study was designed as a feasibility study, we cannot infer about the efficacy of the RISE program on clinical symptoms, even if we observed promising outcomes. The observed improvements in clinical symptoms could be also due to more non-specific factors. Furthermore, the lack of a control groups and the application of strict exclusion criteria limits the generalizability of RISE treatment effects.

## Conclusions

Few clinical trials have examined the effect of psychotherapeutic interventions in patients after suicide attempt within acute psychiatric inpatient settings. A timely intervention after the suicide attempt is highly recommended to reduce the risk of future suicidal behavior. Considering the low dropout rate and the overall positive evaluation, the RISE program was highly accepted in a sample of severely impaired patients, most of whom were multiple attempters. The present study also demonstrated that the levels of suicidal ideations, mental pain, depressive symptoms, and hopelessness decreased significantly after RISE. Since all of these clinical parameters are associated with the risk of future suicidal behavior, a potential suicide-preventive effect of the intervention can be inferred from the present findings. The positive results of the follow-up assessment after 6 months point in the same direction. In addition, RISE treatment increased self-efficacy in patients, which is an important contributor for better coping with future suicidal crises. Thus, present study demonstrate that RISE is a suitable therapy program for the treatment of patients at high risk for suicidal behavior in an acute inpatient setting. Therefore, testing its efficacy in a randomized controlled clinical trial is an important and promising next step.

## Data availability statement

The original contributions presented in the study are included in the article/supplementary materials, further inquiries can be directed to the corresponding author.

## Ethics statement

The studies involving human participants were reviewed and approved by Friedrich-Schiller University, Jena. The patients/participants provided their written informed consent to participate in this study.

## Author contributions

GW, TS, UK, SS, and MW contributed to conception, design, and conducting of the study. ML and LB organized the database and performed clinical assessments. GW and LB performed the statistical analysis and wrote the manuscript. All authors contributed to manuscript revision, read, and approved the submitted version.

## Funding

This work was supported by a research grant from the Bundesministerium für Gesundheit (BMG; Federal Ministry of Health, ZMVI1-2517FSB143).

## Conflict of interest

The authors declare that the research was conducted in the absence of any commercial or financial relationships that could be construed as a potential conflict of interest.

## Publisher's note

All claims expressed in this article are solely those of the authors and do not necessarily represent those of their affiliated organizations, or those of the publisher, the editors and the reviewers. Any product that may be evaluated in this article, or claim that may be made by its manufacturer, is not guaranteed or endorsed by the publisher.
